# Supplementary material for: Publishing neural networks in drug discovery might compromise training data privacy
Source: J Cheminform. 2025 Mar 26;17:38. doi: 10.1186/s13321-025-00982-w (PMC11948693; doi:10.1186/s13321-025-00982-w)
Supplement: Supplementary file 1 — (PDF 413 KB) [file 13321_2025_982_MOESM1_ESM.pdf]

Publishing Neural Networks in Drug Discovery Might Compromise Training Data Privacy

A Preprint

Fabian P. Krüger[1,2,3] **Johan Östman**[4] **Lewis Mervin**[5] **Igor V. Tetko**[3] **Ola Engkvist**[1,6]

[1] **AstraZeneca R&D**
Discovery Sciences
Molecular AI
431 83 Mölndal, Sweden

[2] **Technical University of Munich**
TUM School of Computation,
Information and Technology
Department of Mathematics
80333 Munich, Germany

[3] **Helmholtz Munich - Deutsches Forschungszentrum Für Gesundheit Und Umwelt (GmbH)**
Molecular Targets and Therapeutics Center
Institute of Structural Biology
85764 Neuherberg, Germany

[4] **AI Sweden**
41756 Gothenburg, Sweden

[5] **AstraZeneca R&D**
Discovery Sciences
Molecular AI
CB2 0AA Cambridge, UK

[6] **Chalmers University of Technology**
Department of Computer Science
and Engineering
412 96 Gothenburg, Sweden

Supplementary information

Derivation of the baseline TPR at an FPR of 0

In order to find the baseline for the TPR at FPR $= 0$, we calculate the expected value of the probability distribution for assigning memberships without any knowledge about the model. In our experiment, the adversary gets a training data sample with a probability of $\frac{2}{3}$ and a non-training data sample with a probability of $\frac{1}{3}$. For our baseline we chose to have our baseline built on knowing these probabilities, which is a rather conservative approach. Given that it is more likely that a molecule comes from the training data, our baseline adversary will always predict that it was part of the training data. Therefore, the probability distribution for the TPR at FPR $= 0$ is given by

$$P\left(\text{TPR} = \frac{k}{N}\right) = \left(\frac{2}{3}\right)^k \cdot \frac{1}{3}, \tag{1}$$

with k being the number of true positives and N the total number of positives (training data samples). We are interested in the expected value of this distribution:

$$\mathbb{E}[\text{TPR}] = \sum_{k=0}^{N} \frac{k}{N} \left(\frac{2}{3}\right)^k \cdot \frac{1}{3} \tag{2}$$

$$\mathbb{E}[\text{TPR}] = \frac{1}{3N} \sum_{k=0}^{N} k \left(\frac{2}{3}\right)^k \tag{3}$$

Since our number of training samples N is very large (859 for our smallest dataset), this sum is very well approximated by taking the sum to infinity:

$$\mathbb{E}[\text{TPR}] \approx \frac{1}{3N} \sum_{k=0}^{\infty} k \left(\frac{2}{3}\right)^{k} \tag{4}$$

In order to find a computationally tractable solution for this expected value, we use the closed form of the geometric series:

$$\sum_{k=0}^{\infty} x^{k} = \frac{1}{1-x} \tag{5}$$

We differentiate both sides with respect to x:

$$\frac{d}{dx}\left(\frac{1}{1-x}\right) = \sum_{k=0}^{\infty} k x^{k-1} \tag{6}$$

$$\frac{1}{(1-x)^2} = \sum_{k=0}^{\infty} k x^{k-1} \tag{7}$$

Finally, we multiply both sides of the equation by x to get our desired expression:

$$\frac{x}{(1-x)^2} = \sum_{k=0}^{\infty} k x^{k} \tag{8}$$

Substituting $x = \frac{2}{3}$:

$$\sum_{k=0}^{\infty} k \left(\frac{2}{3}\right)^{k} = \frac{\frac{2}{3}}{\left(1-\frac{2}{3}\right)^{2}} = 6 \tag{9}$$

With this solution, we can now calculate the expected TPR at FPR $= 0$:

$$\mathbb{E}[\text{TPR}] \approx \frac{1}{3N} \cdot 6 = \frac{2}{N} \tag{10}$$

This is the baseline for our TPR in our experiments. Significantly higher values show that it is possible to gather information about the training data from the neural network.

Figures

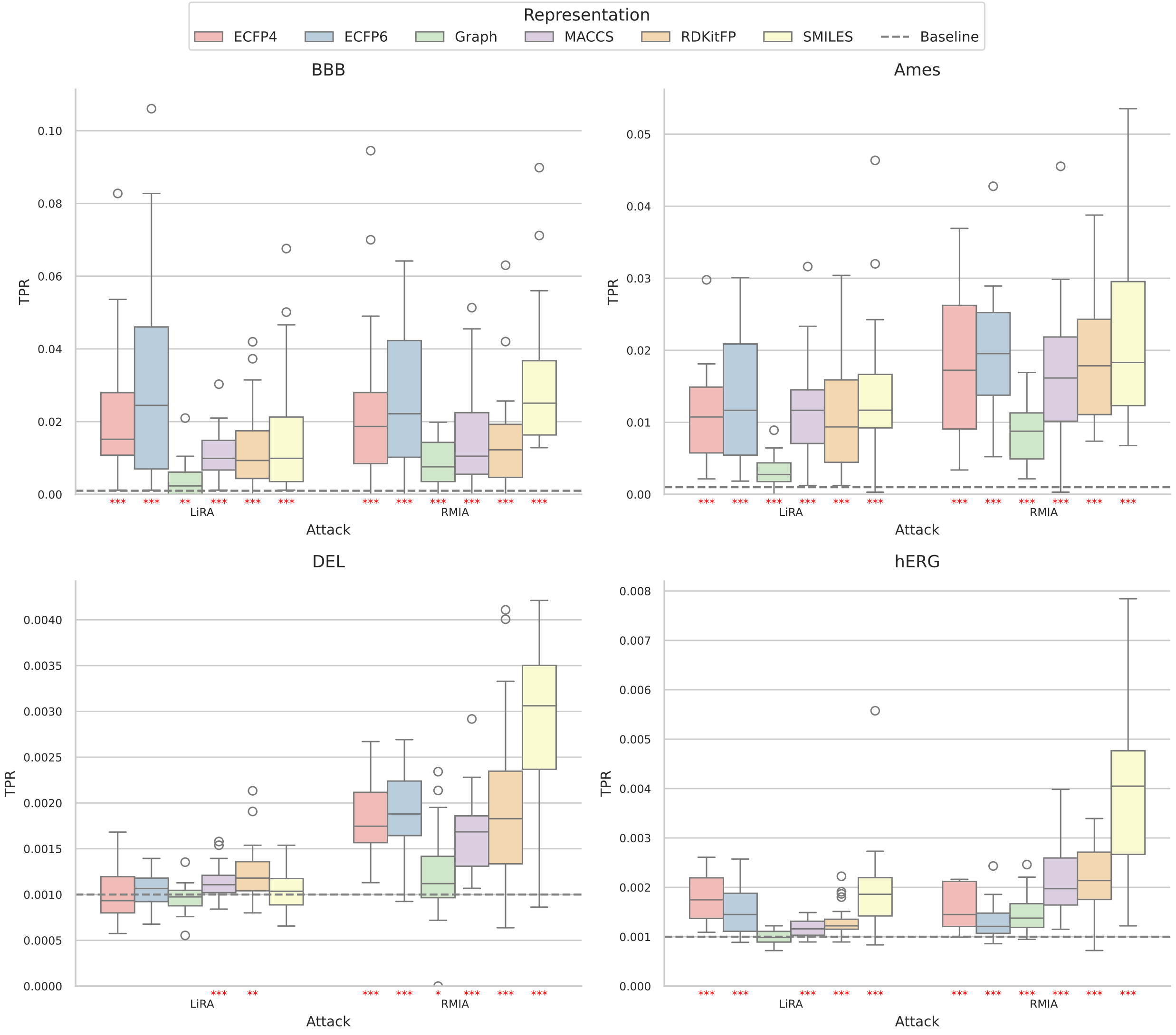

Figure 1: True positive rates for identifying training data molecules at a false positive rate of 10^{-3}. The distributions of 20 experimental repetitions are shown for each representation and dataset, for both the likelihood ratio attack (LiRA) and the robust membership inference attack (RMIA). Distributions with significantly higher true positive rates than the baseline are indicated by red stars. A single star represents a p-value less than 0.05, two stars represent a p-value less than 0.01, and three stars represent a p-value less than 0.001. Training dataset sizes (total amount of positives) are: 859 molecules for the blood-brain barrier permeability dataset; 3,264 for the Ames mutagenicity prediction dataset; 48,837 for the DNA-encoded library enrichment dataset; and 137,853 for the hERG channel inhibition dataset.

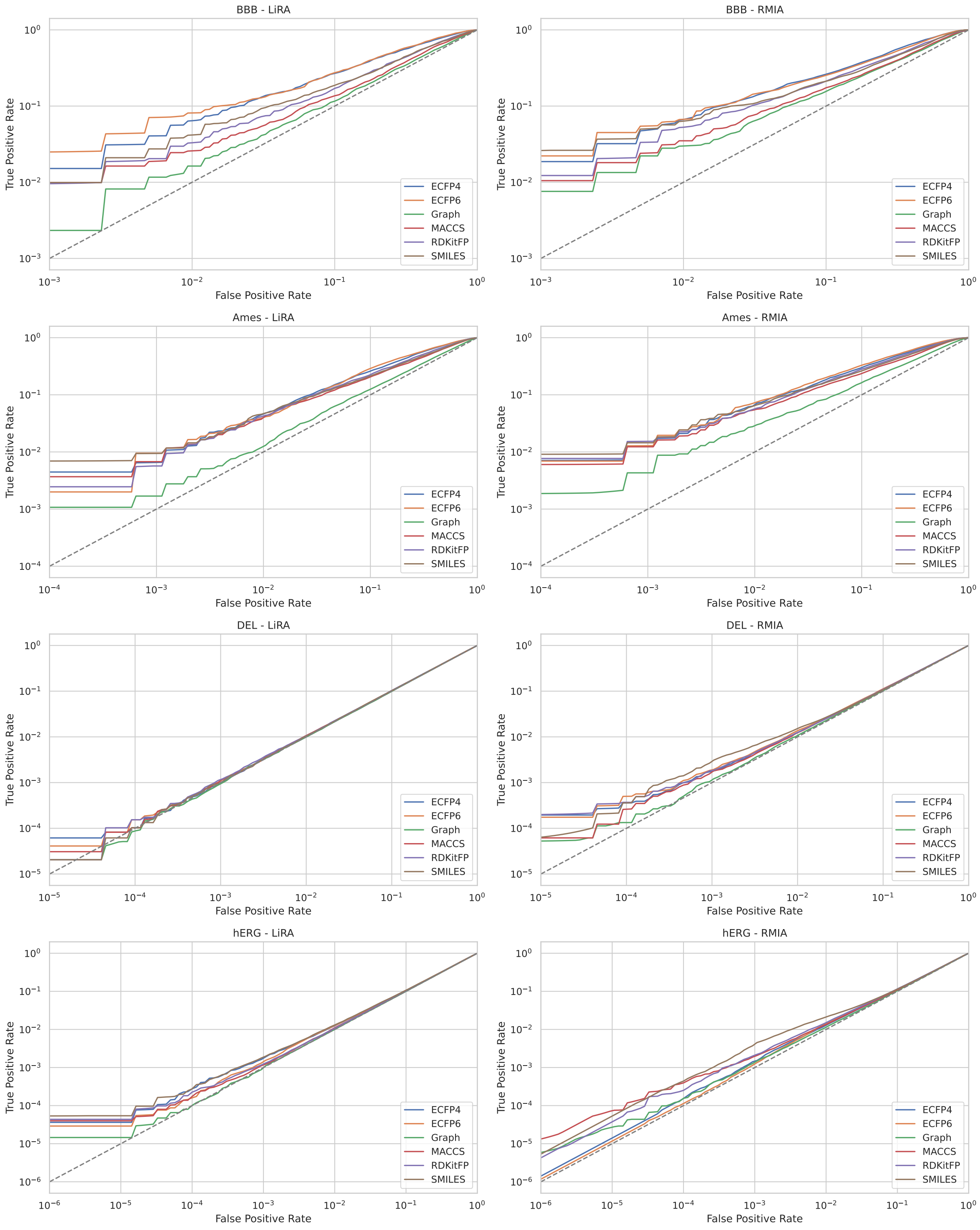

Figure 2: ROC curves for identifying training data molecules. Results are displayed for both the likelihood ratio attack (LiRA) and the robust membership inference attack (RMIA). The dashed line indicates the performance of random guessing. Training dataset sizes are: 859 molecules for the blood-brain barrier permeability dataset; 3,264 for the Ames mutagenicity prediction dataset; 48,837 for the DNA-encoded library enrichment dataset; and 137,853 for the hERG channel inhibition dataset.

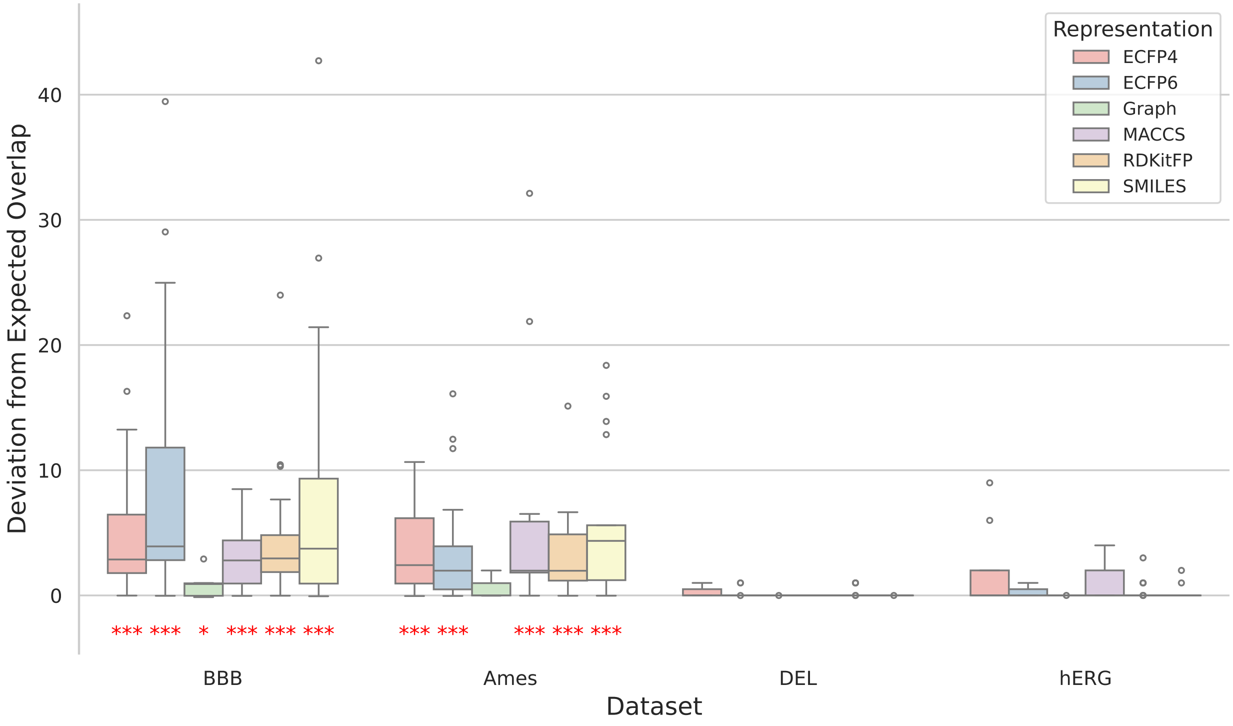

Figure 3: Deviations from the expected overlap of uncorrelated attacks. For each of our 20 experiment repetitions, we calculated the amount of molecules that were identified by both the likelihood ratio attack (LiRA) and the robust membership inference attack (RMIA) for each dataset and representation. We also calculated how many molecules would have been identified by both attacks if they were completely uncorrelated. This amount depended on the sizes of the sets of the identified molecules in each repetition. The figure shows the distribution of deviations from the expected overlap (for uncorrelated attacks) to the observed overlap. Distributions significantly greater than 0 are marked with red stars. A single star represents a p-value less than 0.05, two stars represent a p-value less than 0.01, and three stars represent a p-value less than 0.001.

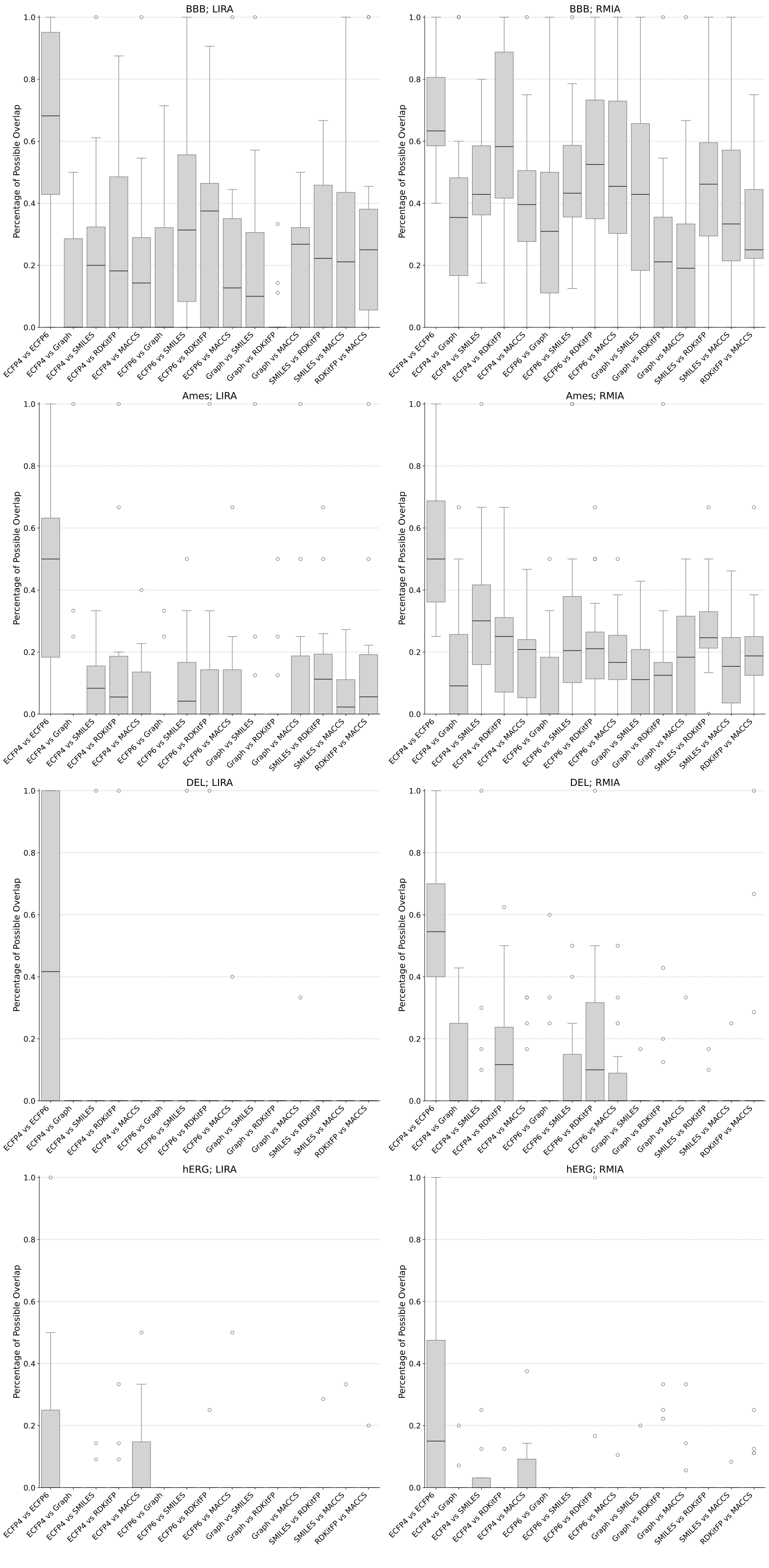

Figure 4: Overlap of identified molecules between models trained on different molecular representations of the same training data. The distribution displays the amount of overlap in our 20 experiment repetitions.

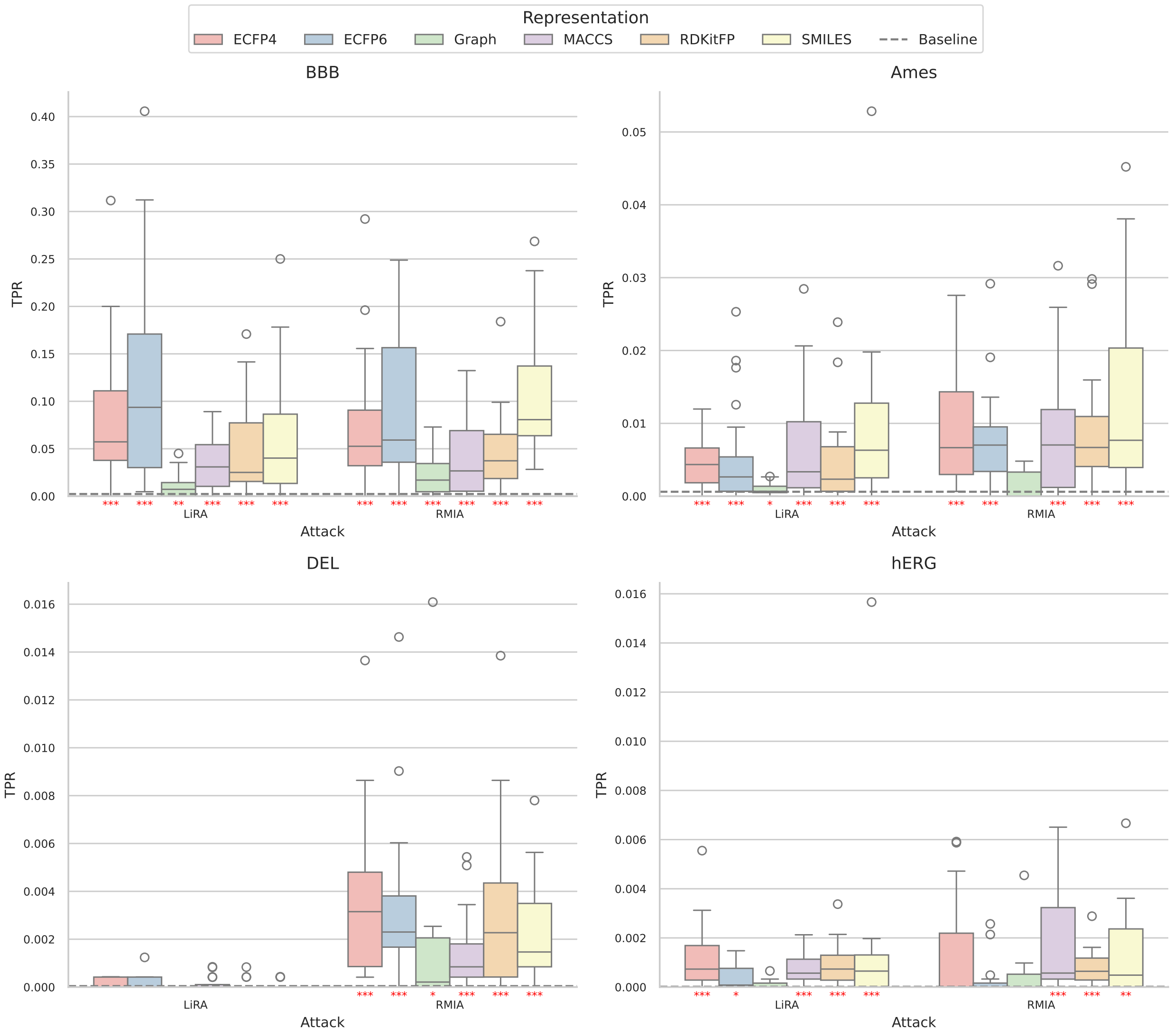

Figure 5: True positive rates for identifying training data molecules of the minority class at a false positive rate of 0. The distributions of 20 experimental repetitions are shown for each representation and dataset, for both the likelihood ratio attack (LiRA) and the robust membership inference attack (RMIA). The baseline for each dataset is 2 divided by the amount of minority class molecules in the training data. Distributions with significantly higher true positive rates are indicated by red stars. A single star represents a p-value less than 0.05, two stars represent a p-value less than 0.01, and three stars represent a p-value less than 0.001.

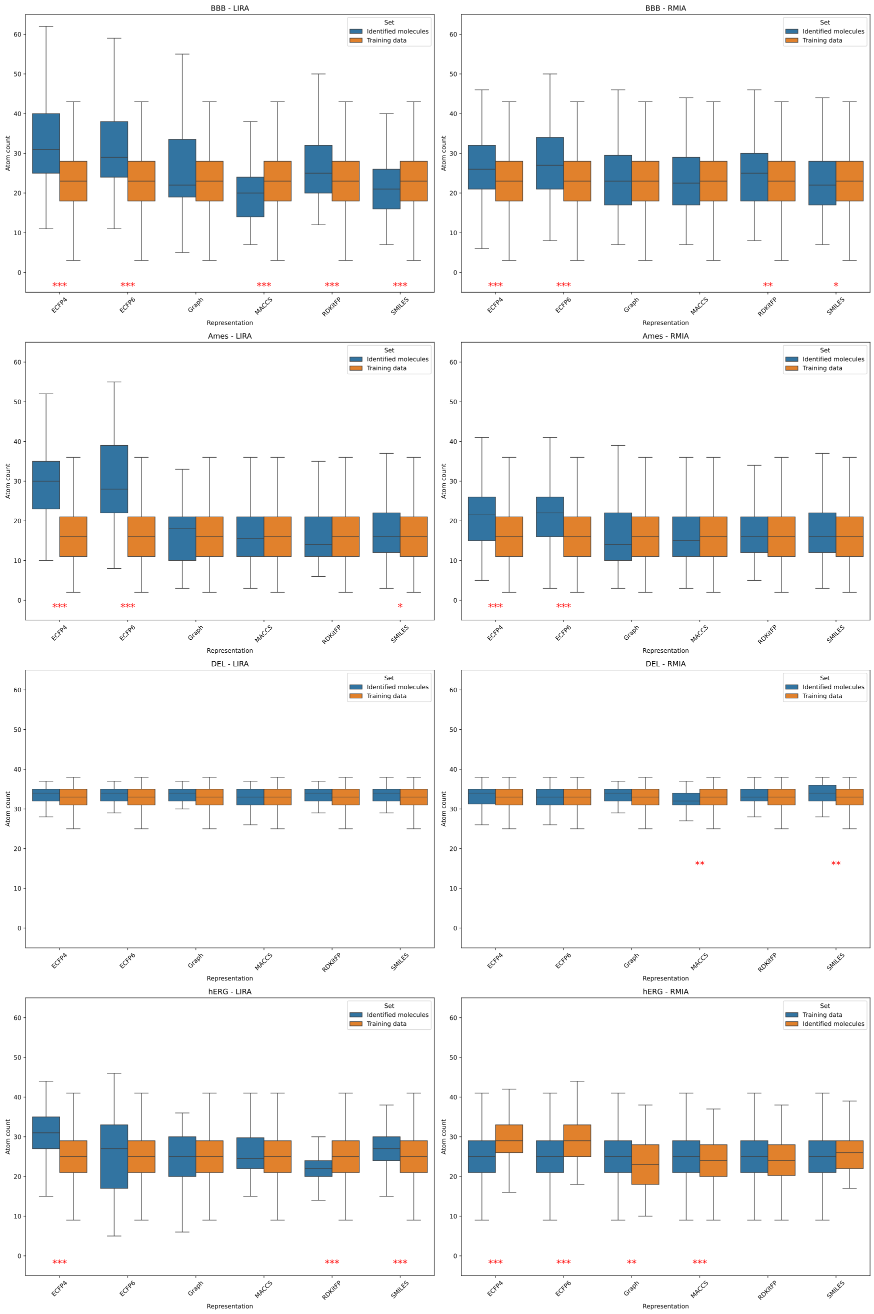

Figure 6: Distributions of molecular sizes, in terms of atom counts, are shown for the chemical structures identified at an FPR of 0 and for the corresponding training data. The data represents the aggregated results from 20 experimental repetitions. Significant differences between distributions are marked with red stars: one star for a p-value less than 0.05, two stars for a p-value less than 0.01, and three stars for a p-value less than 0.001.

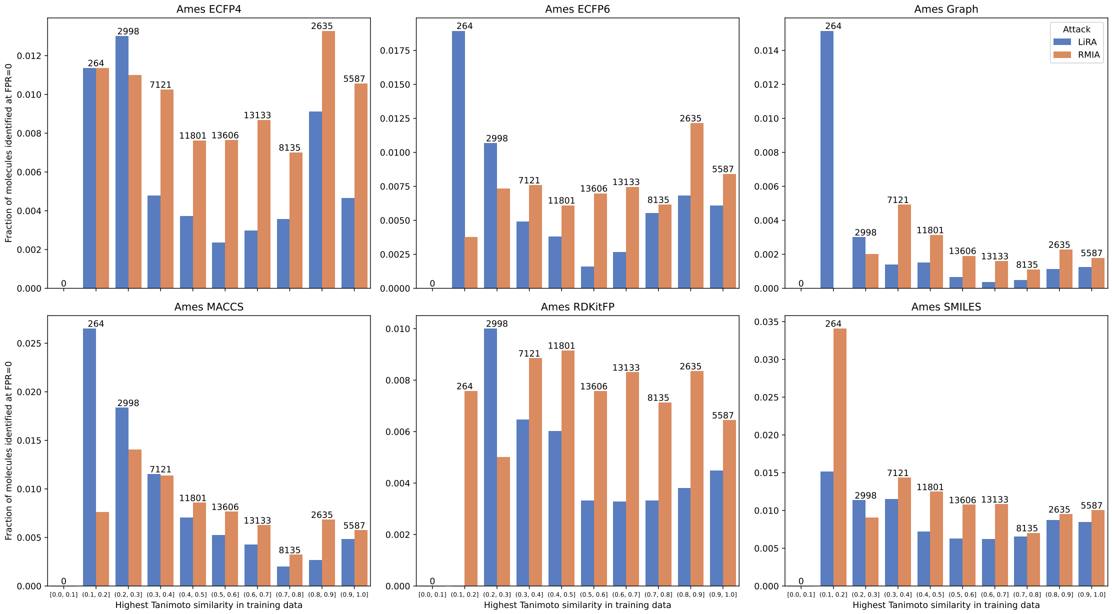

Figure 7: **Nearest-neighbor similarity and privacy vulnerability in the Ames mutagenicity dataset.** Grouped bar plots show, for each molecule representation (ECFP4, ECFP6, Graph, MACCS, RDKitFP, and Transformer-based SMILES matrix), the fraction of molecules that could be identified by LiRA (blue) and RMIA (orange) in the Ames training dataset. Molecules were binned into deciles based on their **highest** (nearest-neighbor) Tanimoto similarity in the training data, and the y-axis reports the fraction of identified molecules identified within each bin.

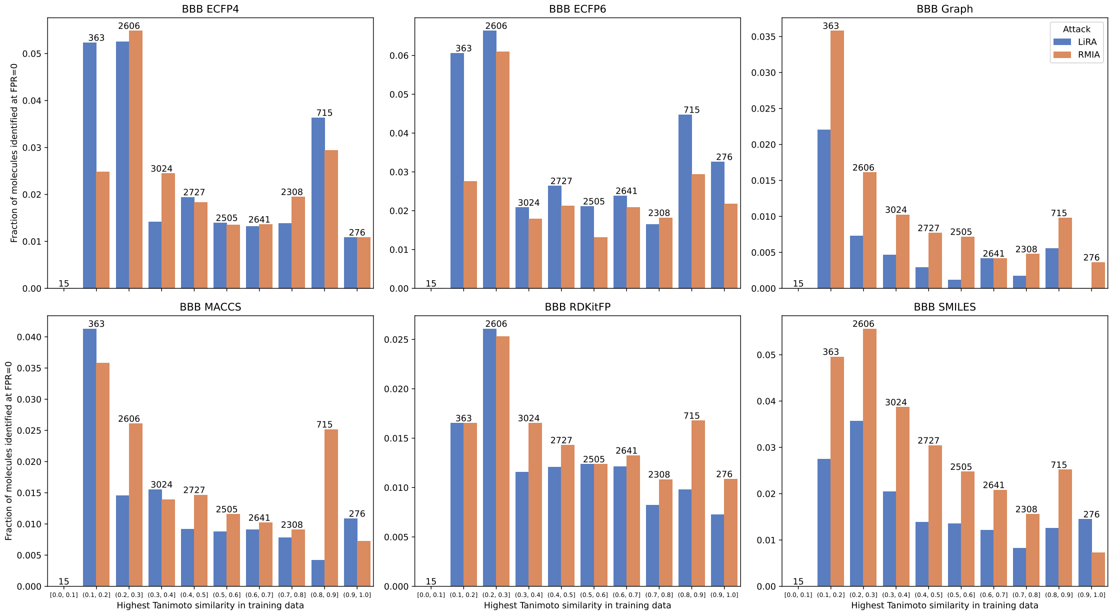

Figure 8: **Nearest-neighbor similarity and privacy vulnerability in the Blood-Brain Barrier crossing (BBB) dataset.** Grouped bar plots show, for each molecule representation (ECFP4, ECFP6, Graph, MACCS, RDKitFP, and Transformer-based SMILES matrix), the fraction of molecules that could be identified by LiRA (blue) and RMIA (orange) in the BBB training dataset. Molecules were binned into deciles based on their **highest** (nearest-neighbor) Tanimoto similarity in the training data, and the y-axis reports the fraction of identified molecules identified within each bin.

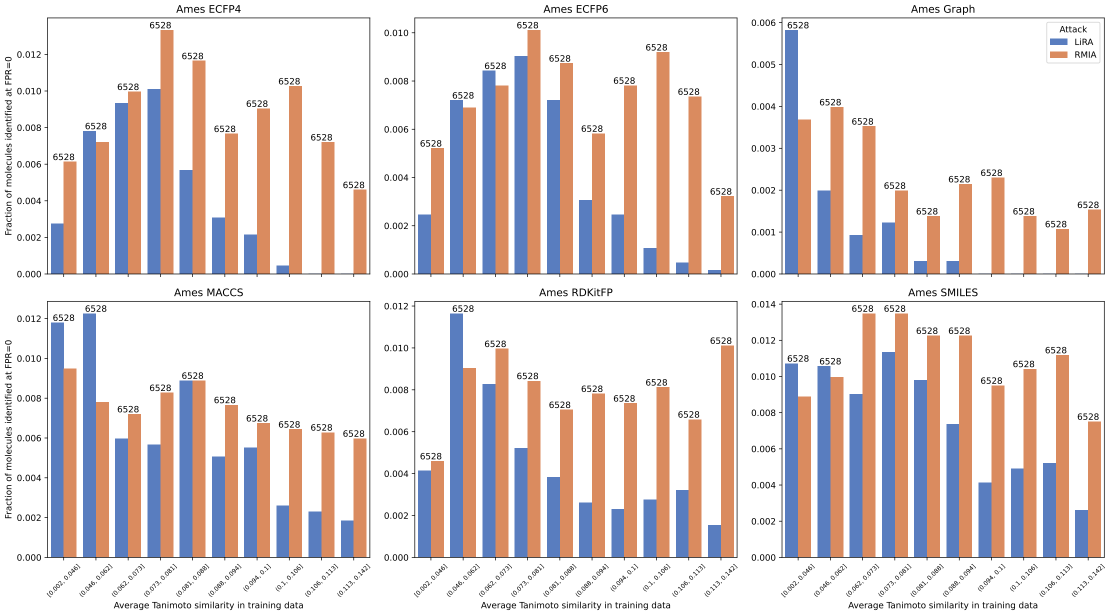

Figure 9: **Average Tanimoto similarity and privacy vulnerability in the Ames mutagenicity dataset.** Grouped bar plots display, for each molecular representation (ECFP4, ECFP6, Graph, MACCS, RDKitFP, and Transformer-based SMILES matrix), the fraction of molecules identified by LiRA (blue) and RMIA (orange) in the BBB training dataset.Molecules were binned into 10 equal-sample (quantile-based) bins based on their **average** Tanimoto similarity in the training data, and the y-axis reports the fraction of identified molecules identified within each bin.

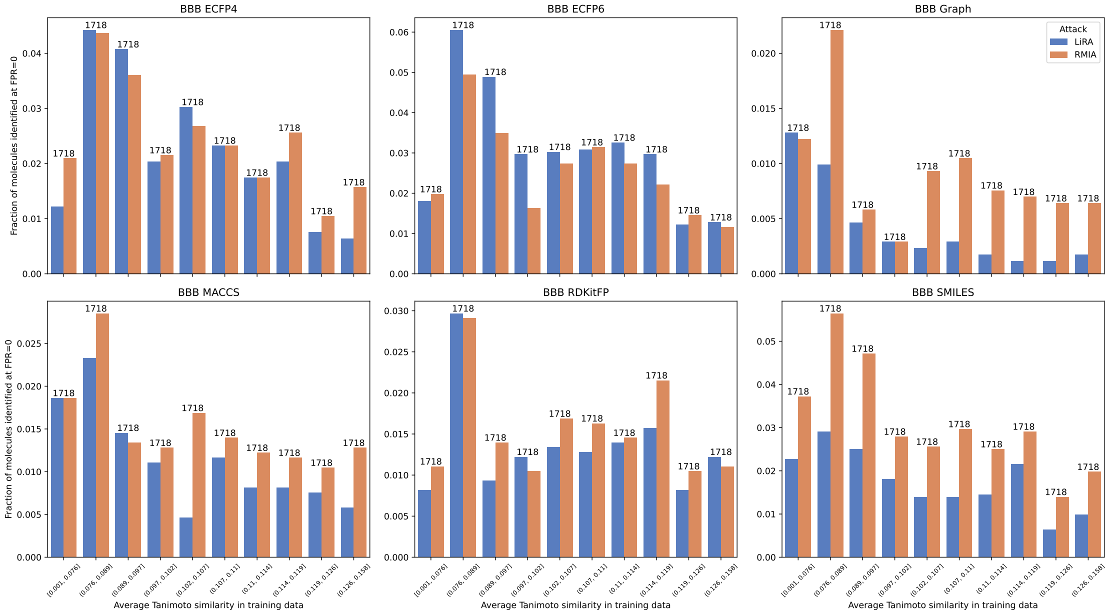

Figure 10: **Average Tanimoto similarity and privacy vulnerability in the Blood-Brain Barrier (BBB) dataset.** Grouped bar plots display, for each molecular representation (ECFP4, ECFP6, Graph, MACCS, RDKitFP, and Transformer-based SMILES matrix), the fraction of molecules identified by LiRA (blue) and RMIA (orange) in the BBB training dataset. Molecules were binned into 10 equal-sample (quantile-based) bins based on their **average** Tanimoto similarity in the training data, and the y-axis reports the fraction of identified molecules identified within each bin.

Tables

Table 1: Hyperparameter search spaces for MLP, MPNN, and CNN models.

Model	Hyperparameter	Range
MLP	Dropout	0.0 – 1.0
	Hidden Dimension	10 – 1000
	Number of Hidden Layers	1 – 5
	Learning Rate	1e-5 – 1e-2
	Weight Decay	1e-6 – 1e-3
MPNN	Message Passing Steps	2 – 6
	Encoder Dropout	0.0 – 0.4
	Encoder Hidden Size	300 – 2400
	Bias in Encoder Layers	{True, False}
	Aggregation Method	{mean, sum, norm}
	Classifier Number of Hidden Layers	1 – 3
	Classifier Hidden Dimension	300 – 2400
	Classifier Dropout	0.0 – 0.4
	Learning Rate	1e-5 – 1e-2
	Weight Decay	1e-6 – 1e-3
CNN	Dropout	0.0 – 1.0
	Learning Rate	1e-5 – 1e-2
	Weight Decay	1e-6 – 1e-3
